# Case report: Surgical valvular pulmonary reconstruction for a previous unreported rheumatic right-sided valve disease with severe pulmonary regurgitation

**DOI:** 10.3389/fcvm.2023.1129529

**Published:** 2023-05-11

**Authors:** Zijing Zhou, Weijie Tang, Xiaokang Tu, Feng Li, Zhonghua Huang, Wancun Jin, Qin Wu, Feng Liu, Chengming Fan

**Affiliations:** ^1^ Department of Pulmonary and Critical Care Medicine, The Second Xiangya Hospital, Central South University, Changsha, China; ^2^ Department of Cardiovascular Surgery, The Second Xiangya Hospital, Central South University, Changsha, China

**Keywords:** cardiac surgery, rheumatic heart disease, pulmonary valve, echocardiography, reconstruction

## Abstract

Rheumatic heart disease (RHD) is a widespread illness in developing countries. RHD causes 99% of mitral stenoses in adults and 25% of aortic regurgitation. However, it only causes 10% of stenoses of the tricuspid valve, and is almost always associated with left-side valvular lesions. Isolated right-side valves are rarely affected, but may result in severe rheumatic pulmonary regurgitation. Herein, we present a case of rheumatic right-sided valve disease with severe pulmonary valve contracture and regurgitation in a symptomatic patient, successfully managed by surgical valvular reconstruction with a tailored bileaflet bovine pericardial patch. The options for surgical approach are also discussed. To our knowledge, the presented rheumatic right-sided valve disease with severe pulmonary regurgitation is the first to be reported in the literature.

## Introduction

In 65%–70% of patients with rheumatic heart disease (RHD), the mitral valve is the most commonly and severely affected, while the aortic valve accounts for 25% of lesions (1). Rheumatic tricuspid valve lesions occur in only 10% of patients and are almost always left-sided. RHD is a widespread illness in developing countries and causes 99% of mitral stenoses in adults, while the pulmonary valve is rarely affected (2). Pulmonary valve stenosis caused by rheumatic disease is quite rare and, when it occurs, it is almost always accompanied by rheumatic lesions of other heart valves. A few cases of rheumatic pulmonary valve disease were reported in 2016 in children under the age of 15 in the tropical zone of India and most of them presented with various degrees of pulmonary stenosis without the involvement of other cardiac valves (3). Severe isolated primary rheumatic tricuspid and pulmonary insufficiency with normal left-side heart valves is extremely rare and almost always accompanied by endocarditis, carcinoid syndrome and trauma (4, 5). Herein, we present a case of a severe rheumatic right-sided valvular regurgitation in a patient without endocarditis, carcinoid syndrome or trauma that was successfully managed by surgical pulmonary valve and trunk reconstruction with a tailored bileaflet bovine pericardial patch. The diagnostic modalities, surgical strategy and choice of operation technique are also discussed.

## Case presentation

A 58-year-old female was referred to our cardiac center complaining of dyspnea on exertion and chest tightness over the last few years, which had gradually worsened in the last 2 weeks with lower limb edema. The patient had no previous history of cardiovascular disease and denied a relevant family history or a history of smoking, alcohol intake or drug abuse. She also denied a history of rheumatic fever. On admission, the patient's body mass index (BMI) and body temperature were 23.3 kg/m^2^ and 36.5°C, respectively. Her blood pressure was 132/86 mmHg with a heart rate and radial pulse rate of 80 bpm.

**Figure 1:**
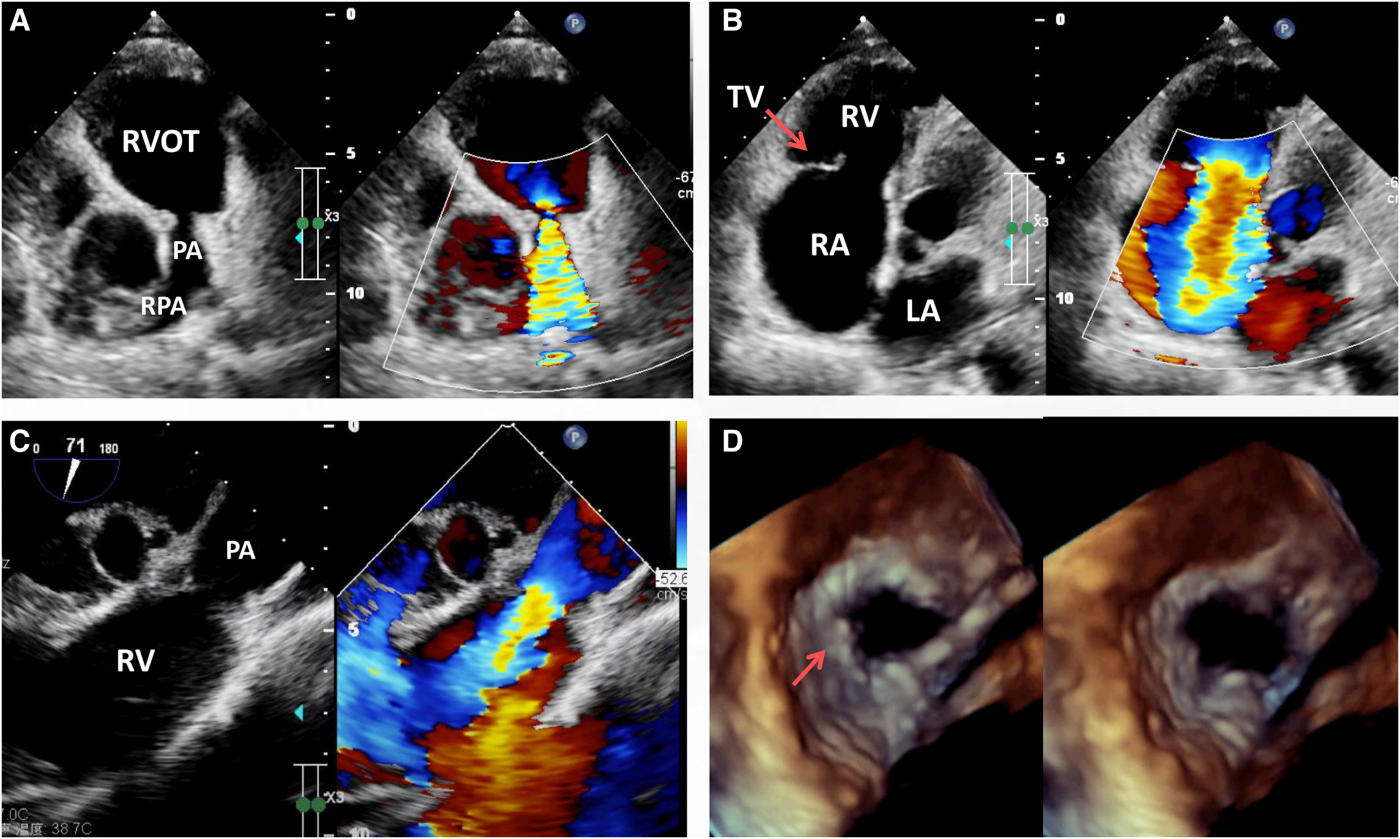
Transthoracic echocardiogram (TTE) pre-operation revealing the presence of severe pulmonary stenosis with the contracture of the pulmonary annulus and valves but with no sign of RVOT stenosis (**A**), RA enlargement, severe tricuspid regurgitation (**B**, arrow) and pulmonary regurgitation (**C**). Transesophageal echocardiography (TEE) immediately before surgery revealing severe pulmonary valvular insufficiency in the cardiac end diastolic period and end systolic period (**D**, arrow). LA, left atrium; PA, pulmonary artery; RA, right atrium; RPA, right pulmonary artery; RV, right ventricle; RVOT, right ventricular outflow tract; TV, tricuspid valve.

Laboratory investigation including routine blood, stool, urine and troponins were all within the normal range. The biochemical examination showed no changes in serum levels of thyroxine, alkaline phosphatase, blood glucose, potassium, sodium, urea, creatinine, transaminase or total bilirubin. NT-pro BNP was 1,372 pg/ml (normal: 25–125 pg/ml) on admission. On physical examination, the patient had good psychomotor development without neurological signs or nystagmus, but peripheral lower limb edema was detected. A severe systolic and diastolic cardiac murmur in the precordial region was audible. ECG showed sinus rhythm with non-specific T-wave abnormalities in the lateral leads. Chest radiograph showed no significant signs of abnormality except for a mild enlarged cardiac silhouette. Coronary arteriography showed normal coronary flow without plaque or stenosis. Transthoracic echocardiogram (TTE) examination revealed the following measurements: left atrium 20 mm, right atrium 56 mm, LV cavity 53 ml (end-diastolic diameter) and 19 ml (end-systolic diameter), aorta 29 mm, pulmonary trunk from 13 to 27 mm with left and right pulmonary artery both 11 mm in diameter (Figure 1A). The pulmonary annulus (4 mm) and leaflets showed severe contraction and stenosis, the Vmax with peak and mean gradient were 3.4 m/s and 45 mmHg, respectively (Figure 1A). Severe tricuspid regurgitation (Figure 1B) and pulmonary regurgitation were also detected (Figure 1C, See Supplementary Video S1). Transesophageal echocardiography (TEE) confirmed the diagnosis (Figure 1D) and showed a short and thick solid ring bulge at the site of the pulmonary annulus (Figure 1D, arrow). Rheumatic right-sided valve disease with severe tricuspid and pulmonary regurgitation was diagnosed before surgery.

**Figure 2:**
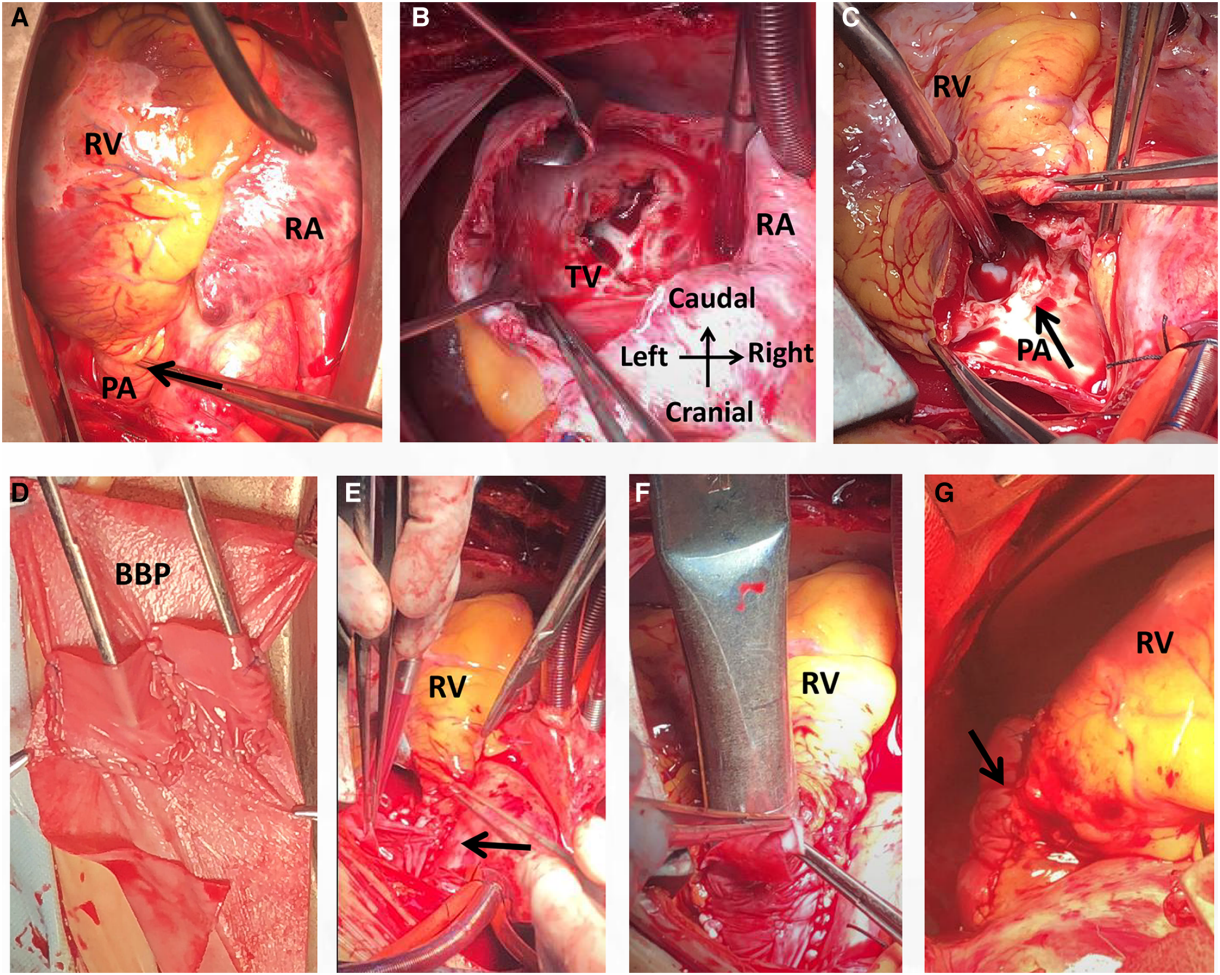
Intraoperative view showing a small pulmonary annulus (**A**), rheumatic tricuspid stenosis with regurgitation (**B**) and pulmonary regurgitation with valvular contracture (**C**). A bileaflet bovine pericardial patch was then tailored (**D**) and used for reconstruction of the pulmonary trunk with the leaflets at the same high level (**E**, arrow). The diameter of the reconstructed pulmonary was 20 mm (**F**). The operation was completed (**G**). BBP, bovine pericardial patch; PA, pulmonary artery; RA, right atrium; RV, right ventricle; RVOT, right ventricular outflow tract; TV, tricuspid valve.

**Figure 3:**
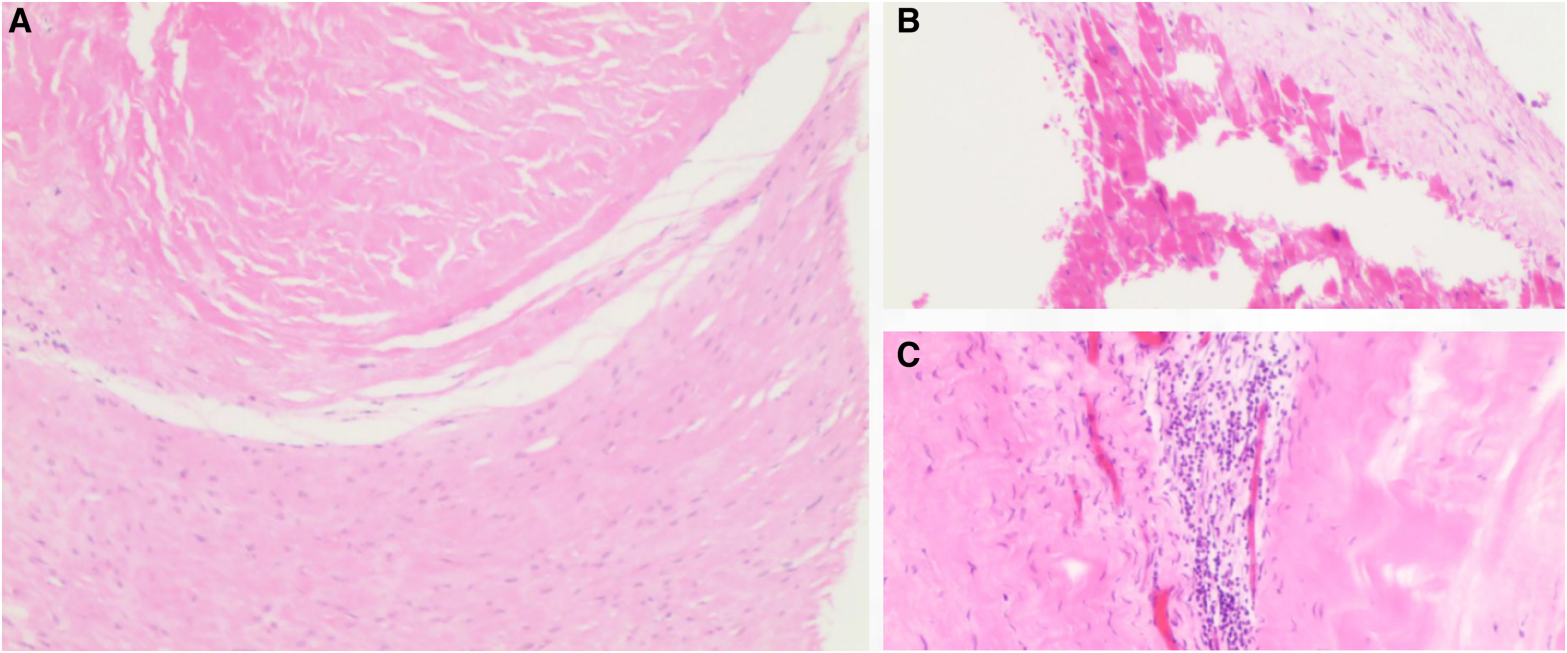
Histopathology of the tricuspid valve showed fibrous tissue hyperplasia with hyalinosis (**A**), mucoid degeneration (**B**) and the infiltrated lymphocytes (**C**). H&E staining, ×10 for panel **A** and ×40 for panel **B,C**.

**Figure 4:**
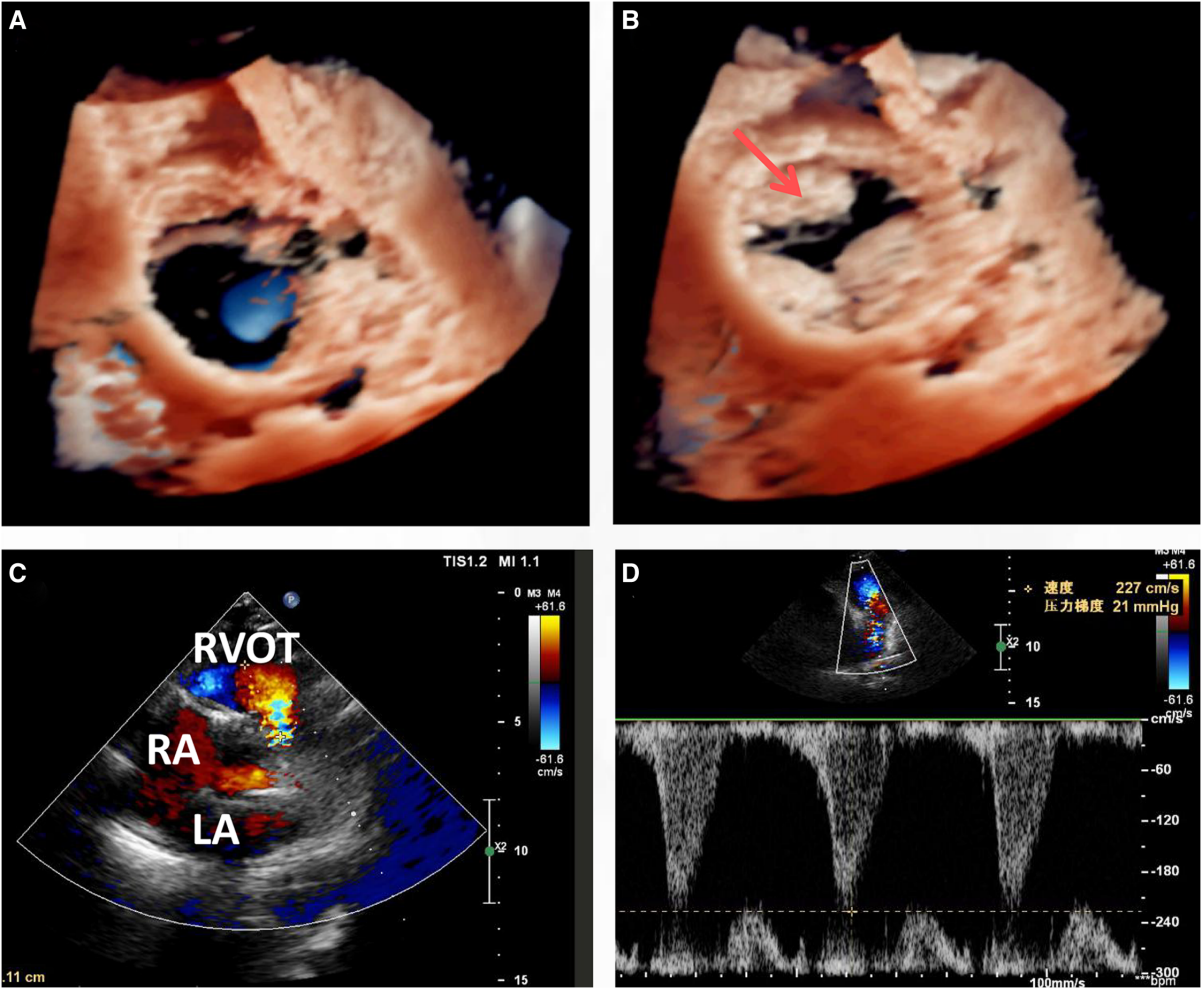
Transthoracic echocardiogram (TTE) at discharge (8 days post-surgery) indicated that the leaflets functioned well in both the cardiac phase of end systolic (**A**) and diastolic (**B**), with only mild regurgitation (**C,D**).

An open-heart operation was performed through a median sternotomy (Figure 2A), and cardiopulmonary bypass was established via routine aortic, superior and inferior vena cava cannulation under mild hypothermia of 30°C. The aortic cross-clamp and theinfusion of any cardioplegic solution were not applied. The tricuspid valve and pulmonary annulus were approached through the right atrium (Figure 2B) and a right ventricular outflow tract (RVOT) incision (Figure 2C), respectively. Valve sizing for tricuspid valve was 37 mm, thus the replacement with a size 29 bioprosthetic valve (Edwards 7300TFX) was performed. Notably, only one residual pulmonary valvular tissue was observed (Figure 2C, arrow), thus a bileaflet (23 mm each) bovine pericardial patch (Figure 2D) was tailored with a running suture and used to reconstruct the pulmonary trunk and the valves (Figure 2E) via a running suture with the leaflets at the same high level (*in situ*, Figure 2E, arrow) using Ozaki's technique (6). Once the reconstructed pulmonary trunk had been enlarged (Figure 2F), the technique was completed (Figure 2G). The reconstructed pulmonary valvular ring was measured with 20 mm in diameter. Hemostasis was then achieved, followed by chest closure. Intraoperative histopathologic examination of the excised specimen revealed fibrous tissue hyperplasia with hyalinosis (Figure 3A), mucoid degeneration (Figure 3B) and a few lymph cells infiltrated without valve calcification, which confirmed the diagnosis of rheumatic valvular heart disease (Figure 3). The operation was uneventful with Cardiopulmonary bypass time of 87 min. The postoperative course was also uneventful. The patient was discharged from the cardiac intensive care unit and from the hospital on the 2nd and 8th postoperative day, respectively. The TTEs performed at discharge detected good functioning of the bioprosthetic valve and the reconstructed pulmonary valves with only mild pulmonary regurgitation (Figure 4). During the 4 months of follow-up, the patient had an uneventful recovery and was symptom-free.

## Discussion and conclusion

Pulmonary valve disease is often congenital, and only rarely do acquired disorders such as carcinoid and rheumatic fever affect the pulmonary valve (7). As with other types of valvular regurgitation, pulmonary valve regurgitation is a well-known valvulopathy that involves leakage of the pulmonary valve in the cardiac diastolic phase that leads to reverse blood flow heading from the pulmonary to the right ventricle. Etiologies of pulmonary regurgitation may be due to the valvular pathology, considered as a primary cause, or due to the dilation of the annulus from pulmonary arterial hypertension or pulmonary artery dilation, considered as a secondary cause (8). Rheumatism is a rare primary cause. Additional causes of pulmonary regurgitation include carcinoid heart disease, trauma and endocarditis (7). It was reported that acute rheumatic endocarditis involving the pulmonary valve may be more common than chronic valvular scarring resulting in clinically recognizable disease (9). However, isolated rheumatic right-sided valve disease with severe pulmonary valve contracture and regurgitation as presented in this case is extremely rare.

Echocardiography, which can be used to observe the valvular shape, size, effect on diastolic cardiac function, relationship with the adjacent tissues, and severity of the valve noninvasively, is currently the preferred diagnostic tool for patients with a suspected valvular disease (10). Mild pulmonary regurgitation detected via echocardiography is quite common and present in up to 78% of people (10). Notably, transthoracic echocardiography (TTE) is deficient in distinguishing the detailed features of various tissues, which was also apparent in the presented case. Thus, transesophageal echocardiography (TEE), which allows better visualization than TTE, is widely used during surgery. In the presented case, the lack of a pulmonary valve was misdiagnosed via TTE; however, the residual valvular tissue was observed via TEE and intraoperatively (Figure 1D arrow and Figure 2C arrow).

Valvular replacement is a traditional strategy for patients with severe rheumatic right-sided valve disease (7, 9). However, there are often anatomic challenges within the RVOT, including a small annulus, that limit replacement options (6). Nonvalved transannular patches are still frequently used because availability of the conduit remains problematic in some countries. This nonvalved patch technique may lead to severe pulmonary regurgitation and volume overload of the right ventricle, leading to right ventricular dilation, dysfunction and finally failure, arrhythmias, and sudden death. Conversely, due to valvular calcification, dysfunction and lack of growth potential, valved conduits are prone to stenosis and insufficiency, especially in young patients (6). Pulmonary valve replacement with polytetrafluoroethylene single leaflet, polytetrafluoroethylene bileaflet and trileaflet valves has been described but generally has not been broadly reproducible and has shown limited long-term competence (11, 12). In the presented case, residual valvular tissue was observed (Figure 1D arrow and Figure 3C arrow), and due to the small annulus, a tailored bileaflet (instead of trileaflet) bovine pericardial patch was used to reconstruct the pulmonary valve and trunk. Given the good functioning of the tailored bileaflet and the residual valvular tissue (Figure 4B), only mild regurgitation was detected during the follow-up period.

Although early technical success of the pulmonary valvular reconstruction with a tailored bileaflet bovine pericardial patch was achieved, the optimal leaflet material remains under debate. A larger sample size and long-term follow-up are needed.

## Data Availability

The raw data supporting the conclusions of this article will be made available by the authors, without undue reservation.
