# Supplementary material for: Case report: Surgical valvular pulmonary reconstruction for a previous unreported rheumatic right-sided valve disease with severe pulmonary regurgitation
Source: Front Cardiovasc Med. 2023 May 11;10:1129529. doi: 10.3389/fcvm.2023.1129529 (PMC10213930; doi:10.3389/fcvm.2023.1129529)
Supplement: Supplementary file 1 [file Table1.docx]

| TTE | LA (mm) | LV (mm) | AO/PA | EDV (ml) | ESV (ml) | EF (%) | FS | SV (ml) | RA (mm) | RV (mm) | RVOT (mm) | CO (l/min) | mPA (mm) | MPAP (mmHg) | PV ring (mm) | PG (mmHg) | VPA (m/s) | AT (ms) | TAPSE (mm) | TV ring (mm) |
| --- | --- | --- | --- | --- | --- | --- | --- | --- | --- | --- | --- | --- | --- | --- | --- | --- | --- | --- | --- | --- |
| Pre-op | 20 | 36 | 29/13 | 53 | 19 | 65 | 35 | 34 | 59 | 45 | 36 | 3.1 | 13-27 | 20 | 12 | 45 | 3.4 | 120 | 21 | 41 |
| Post-op | 24 | 40 | 22/23 | 69 | 24 | 64 | 35 | 44 | 30 | 30 | 35 | 3.8 | 23 | 15 | - | 21 | 2.2 | 100 | - | - |

TTE data from Pre- and Post-operation

Note: op, operation; LA, left atrium; LV, left ventricle; RA, right atrium; RV, right ventricle; RVOT, right ventricular outflow tract; AO, ascending aorta; PA, pulmonary artery; EDV, end-diastolic volume (LV); ESV, end-systolic volume; EF, ejection fraction; FS, fraction shortening; SV, stroke volume; CO, cardiac output; VPA, velocity at PA; PG, pressure gradient; AT, acceleration time; MPAP, mean pulmonary artery pressure; TAPSE, tricuspid annular plane systolic excursion; mPA, main PA; TV, tricuspid valve.
